# Neuropathy of the suprascapular and axillary nerves in rotator cuff arthropathy: a prospective electrodiagnostic study

**DOI:** 10.1007/s00264-024-06130-7

**Published:** 2024-03-13

**Authors:** Yaiza Lopiz, Alberto Rodríguez-González, Susana Martín-Albarrán, Raul Herzog, Carlos García-Fernández, Fernando Marco

**Affiliations:** 1 Shoulder and Elbow Unit, Department of Traumatology and Orthopaedic Surgery, Clínico San Carlos Hospital, , 5º Planta, Ala Sur. Calle Profesor Martín Lagos S/N, 28004 Madrid, Spain; 2 Department of Surgery, Complutense University, Madrid, Spain; 3 Clinical Neurophysiology Department, Clínico San Carlos Hospital, Madrid, Spain

**Keywords:** Rotator cuff tear arthropathy, RSA, Neurological injury, Axillary nerve injury, Suprascapular nerve injury, Electromyographic study

## Abstract

**Purpose:**

Prevalence of axillary (AN) and/or suprascapular (SSN) neuropathy in rotator cuff tear arthropathy (RCTA) is unknown. We aimed to prospectively evaluate for preoperative neurodiagnostic abnormalities in order to determine their prevalence, location, and influence on reverse shoulder arthroplasty (RSA) outcomes.

**Methods:**

Patients who underwent RSA for RCTA were prospectively included. An electromyography and nerve conduction study were performed pre and post-surgery. Clinical situation: VAS, Relative Constant-Murley Score (rCMS) and ROM over a minimum of two years follow-up.

**Results:**

Forty patients met the inclusion criteria; mean follow-up was 28.4 months (SD 4.4). Injuries in RCTA were present in 83.9% (77.4% in AN and 45.2% in SSN). There were no differences on preoperative VAS, ROM, and rCMS between patients with and without preoperative nerve injuries. Four acute postoperative neurological injuries were registered under chronic preoperative injuries. Six months after RSA, 69% of preoperative neuropathies had improved (82.14% chronic injuries and 77.7% disuse injuries). No differences in improvement between disuse and chronic injuries were found, but patients with preoperative neuropathy that had not improved at the postoperative electromyographic study at six months, scored worse on the VAS (1.44 vs 2.66; *p* .14) and rCMS (91.6 vs 89.04; *p* .27).

**Conclusions:**

The frequency of axillary and suprascapular neuropathies in RCTA is much higher than expected. Most of these injuries improve after surgery, with almost complete neurophysiological recovery and little functional impact on RSA. However, those patients with preoperative neuropathies and absence of neurophysiological improvement six months after surgery have lower functional results.

## Introduction

The prevalence of axillary (AN) and/or suprascapular (SSN) neuropathies in rotator cuff arthropathy (RCTA) is largely unknown [1], as is their possible impact on functional results after reverse shoulder arthroplasty (RSA).

SSN injury has historically been considered rare and its diagnosis was made by exclusion criteria. Apart from post-surgery, these injuries have been described in isolated cases in high-level athletes [2]. Reported axillary nerve injuries are limited to some clinical series related to a traumatic event (glenohumeral dislocation, repetitive microtrauma, or fractures) [3].

The exact mechanism that causes preoperative neuropathies in massive rotator cuff tears or RCTA is not clear. It is unknown whether preoperative AN injury is a cause or consequence of RCTA [4]. In relation to the presence of SSN injuries, the most accepted theory is preoperative traction injury from a massive rotator cuff tear [5].

The studies that do consider these injuries do not clearly define the neurophysiological diagnostic criteria used, which makes it difficult to compare results.

The aim of the present study is to analyse the prevalence of preoperative neurological injuries in the setting of RCTA and their influence on RSA outcome or implant failure.

The main hypothesis of the present study is that AN and SSN nerve injuries in RCTA are more prevalent than expected and their presence could be associated with worse functional outcome after RSA.

## Material and methods

Approved from our Institutional Review Board (protocol code C.P.-C.I.14/512-E). The inclusion criteria were patients with RCTA Hamada grades II–V who underwent RSA. We excluded patients who had any previous surgery on the affected or contralateral shoulder or any comorbidity that might result in neuropathy (e.g. diabetes mellitus, alcoholism, or demyelinating disorders).

### Functional evaluation

Pre- and postoperative functional outcomes (at 1, 3, 6, and 12 months, and yearly from then on) were measured with VAS scale, ROM, and relative Constant-Murley Score (rCMS). Because shoulder function and strength differ by sex and age, we used the rCMS based on the normalised values of the CMS [5]. Clinical evaluation was performed by an independent surgeon who was not involved in the original surgery.

### Electrodiagnostic study

**Fig. 1:**
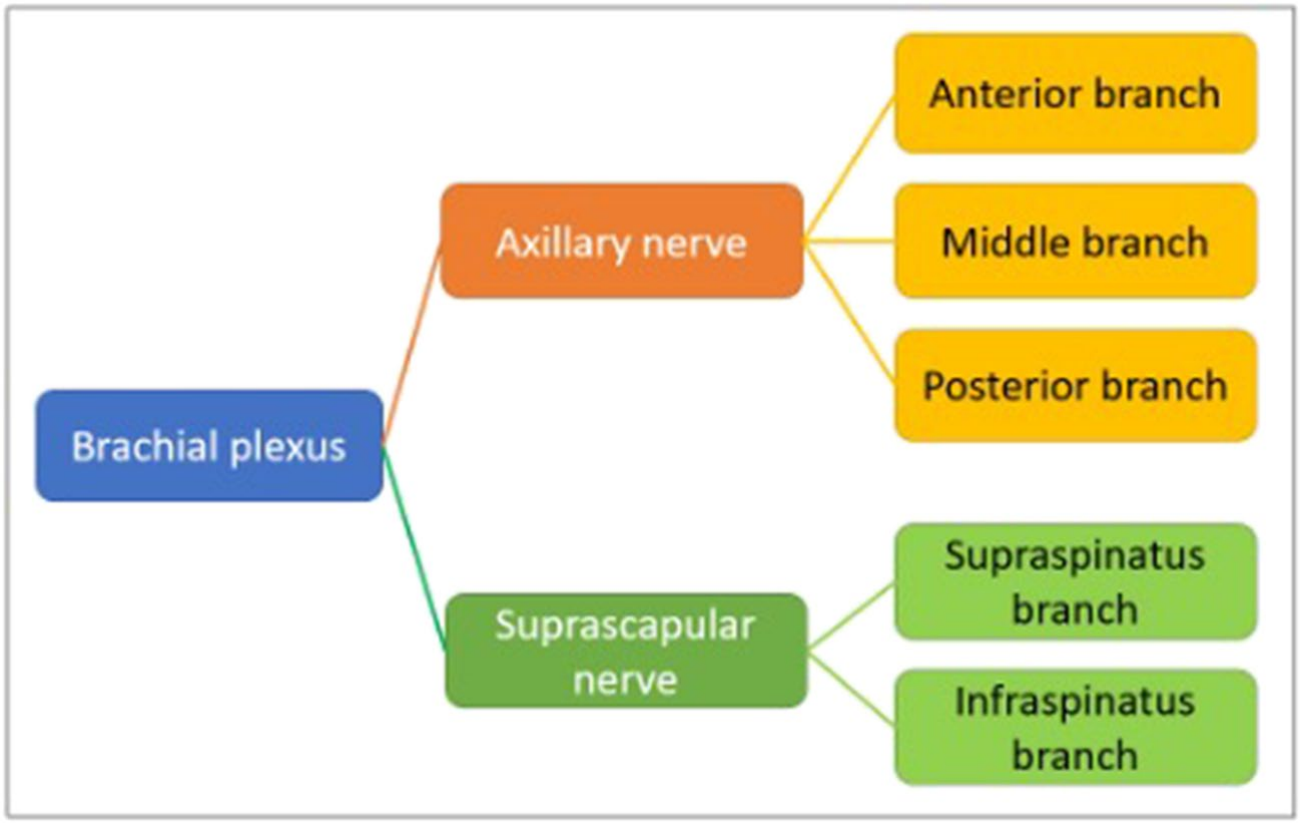
Nervous structures analysed

Electrodiagnostic evaluation was performed preoperatively and at three and six months postoperatively by a single neurophysiologist (S.M.A.), with expertise in EMG of the upper limb, who was blinded to the clinical information. The examination consisted of a motor study of the SSN and motor and sensory studies of the axillary nerve. The middle branch of the AN was considered as the motor fibres of the anterior branch that innervate the middle portion of the deltoid (Fig. 1). The values in the contralateral unaffected shoulder were used as controls. Abnormal findings were documented when the difference to the contralateral healthy side was > 50%. This procedure classified the neurologic state into three types: normal, chronic, and disuse injuries.

In chronic axonal injury, the number of motor units is reduced, therefore will register a decrease in motor unit recruitment during maximum muscular effort and increase in the number of polyphasic motor unit action potentials (reinnervation signs), as well as an increase in their duration and amplitude. In disuse injury there are no denervation or reinnervation signals. There is only a decrease in the number of motor units recruited during maximum muscular effort and a reduction in the amplitude of compound muscle action potentials.

### Radiologic evaluation

**Fig. 2:**
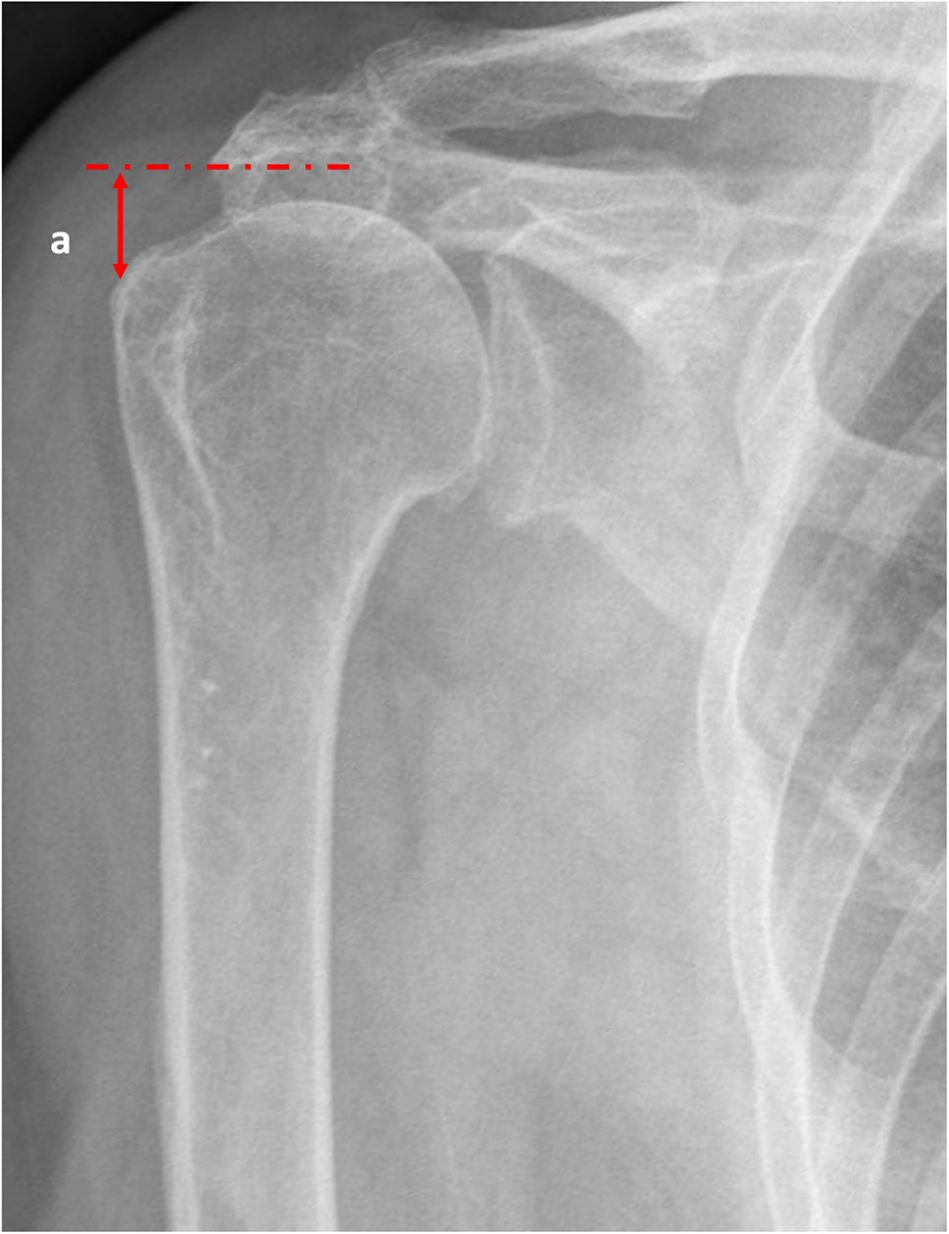
Acromio-greater tuberosity distance measurement

In order to determine whether there was a relationship between superior migration of the humeral head and the presence of preoperative neuropathies (chronic or disuse), the acromion-greater tuberosity (AT) distance was determined. Using complete pre- and postoperative true anteroposterior radiographs of the glenohumeral joint in neutral rotation, the distance from the inferolateral tip of the acromion to the most prominent superolateral aspect of the greater tuberosity was measured (Fig. 2).

### Surgical technique

All surgeries were performed by one of the three senior shoulder surgeons (Y.L., C.G.-F., F.M.) and at least two of them were present at every surgery (the surgical technique has been previously described^3^). The implants employed were Delta Xtend reverse shoulder arthroplasty (54.8%) (DePuy-Johnson & Johnson, Warsaw, IN, USA) and Lima SMR (45.2%) (Lima LTO, San Daniele del Friuli, Italy).

### Statistical analysis

Qualitative variables are presented with their frequency distribution and percentages. The mean, SDs, and ranges are reported for the continuous variables. Continuous variables that showed a skewed distribution are summarised with median and interquartile ranges (IQRs). We used the Mann–Whitney *U* test to compare scores between normal continuous variables and dichotomic variables. We evaluated the association between qualitative variables with the *X*^2^ test or Fisher’s exact test. A comparison of continuous variables with qualitative variables with > 2 categories was performed by analysis of variance. A Kruskal–Wallis nonparametric test was used for variables with skewed distribution. Clinical parameters of interest were compared with the 2-tailed Wilcoxon test or the X^2^ test, when appropriate. Level of significance was set at *p* < 0.05.

## Results

### Epidemiological results

**Fig. 3:**
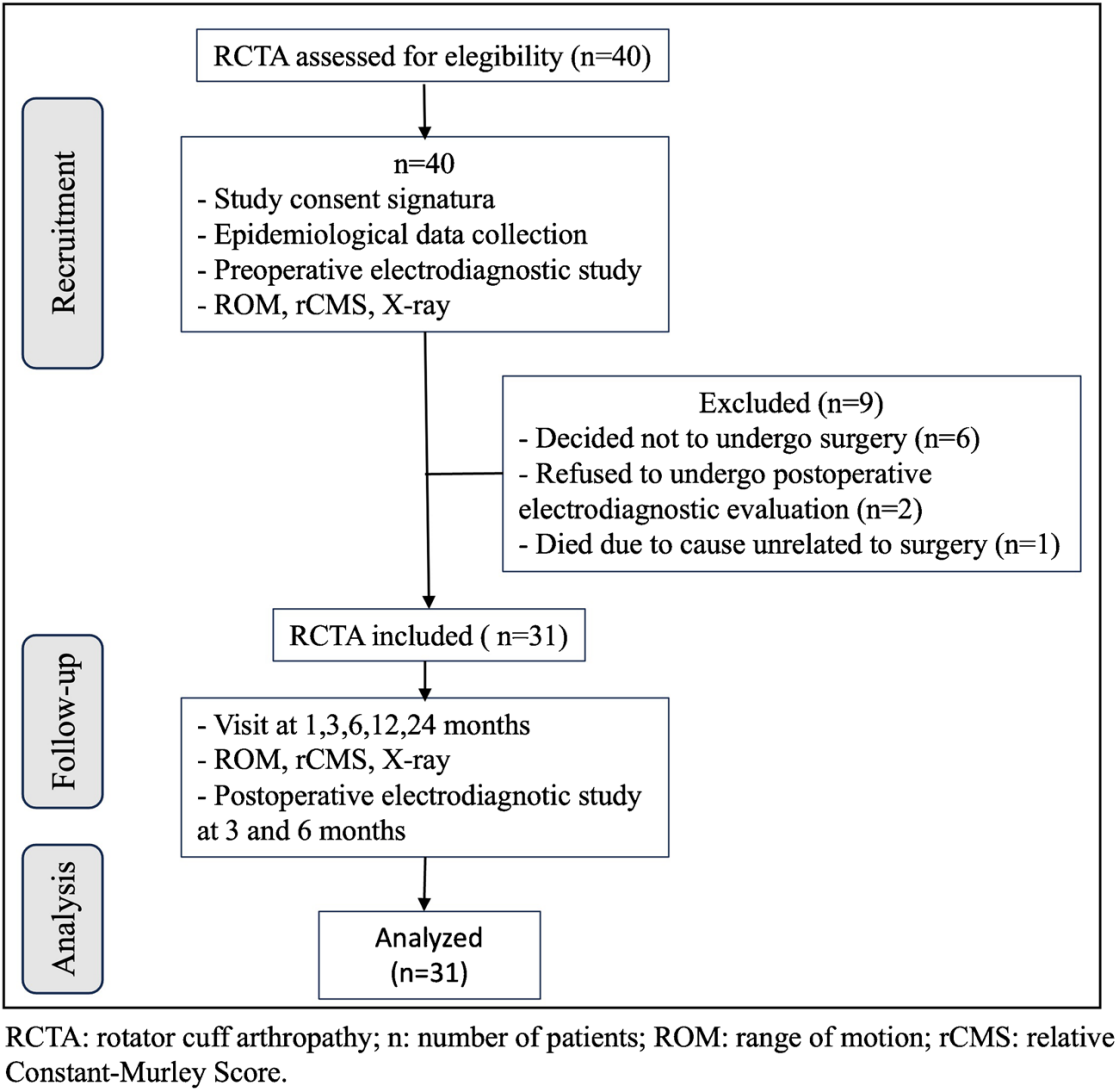
Study flowchart (*n*, number of patients; ROM, range of motion; rCMS, relative Constant-Murley Score)

Forty patients met the inclusion criteria. Nine patients (22.5%) were lost to follow-up, six of them (15%), after being enrolled in the study and with the preoperative electrodiagnostic study performed, decided not to undergo surgery for personal reasons. Therefore, the final sample was reduced to 31 patients with a mean follow-up of 28.4 months (SD 4.4; min. 24 and max. 36). The flowchart is described in Fig. 3, and the demographic data and global functional outcome are summarised in Table 1.

**Table 1:** Demographic data and overall results of the sample at 24 months of follow-up (m, months; No., number; SD, standard deviation; RSA, reverse shoulder arthroplasty; preOp, preoperative; postOp, postoperative; VAS, visual analogue scale; rCMS, relative Contant-Murley Score; Flex., flexion; Abd., abduction; Int. R., internal rotation)

	No. (%) or mean and SD	
Sex		
Female	29 (93.5)	
Male	2 (6.5)	
Age (years)	78 ± 5.4	
Side		
Right	24 (77.4)	
Left	7 (22.6)	
RSA in dominant limb		
Yes	26 (83.9)	
No	5 (16.1)	
HAMADA		
II	21 (67.8)	
III	4 (12.9)	
IV	4 (12.9)	
V	2 (6.4)	
Follow-up	28.4 months	(min. 24/max. 36)
VAS (preOp-PostOp)	8.19 (1.2)–2.10 (2.1)	*p* < .001
rCMS (preOp-PostOp)	39.4 (10.7)–87.1 (8.7)	*p* < .001
Flex. (preOp-PostOp)	79º (27.8)–128º (32.8)	*p* < .001
Abd. (preOp-PostOp)	71º (21.6)–117º (33.5)	*p* < .001
Int. R. (preOp-PostOp)	2.7 (1.0)–3.5 (1.3)	*p* < .001

### Electrodiagnostic evaluation

The prevalence of preoperative neuropathies was 84%, with 77.4% (24 injuries) in AN and 45.2% (14 injuries) in SSN. The most prevalent injuries affecting AN were disuse injuries of the posterior branch (64.5%) and chronic injuries of the anterior branch (35.5%). Regarding the SSN, the most prevalent injuries were disuse injuries of the infraspinatus branch (16%) and chronic injuries of the supraspinatus branch (16%).

Six months after RSA, pre-surgical injuries showed an improvement greater than 69%, except for the anterior and posterior branches of the AN, with an absence of improvement of 40% and 30%, respectively. Regarding the type of injury, 82% chronic injuries (23/28 patients) and 77.7% disuse injuries (28 of 36) improved six months after surgery. We did not find any relationship between the presence of preoperative injury and the onset of acute postoperative injury (*p* 0.631). Table 2 summarises the affected branch of each nerve and its evolution in EMG six months after surgery.

**Table 2:** Preoperative nerve injuries and their improvement 6 months after surgery (preOp, preoperative)

Nerve		Nerve injury			Improvement		
	Branch	Normal (cases)	Chronic (cases)	Disuse (cases)	Improvement (cases)	Not improv. (cases)	Acute over preOp injuries
Axillary	Anterior	51.6% (16)	35.5% (11)	12.9% (4)	46.7% (7)	40% (6)	13.3% (2)
	Middle	71% (22)	19.4% (6)	9.6% (3)	77.8% (7)	22.2% (2)	
	Posterior	25.8% (8)	9.7% (3)	64.5% (20)	69.6% (16)	30.4% (7)	
Suprascapular	Supraspinatus	71% (22)	16.1% (5)	12.9% (4)	88.9% (8)	11.1% (1)	
	Infraspinatus	74.2% (23)	9.7% (3)	16.1% (5)	50% (4)	25% (2)	25% (2)

### Analysis of the AT distance and the preoperative neurological injury

The mean global pre-surgical AT distance was 7.34 mm (SD 5.3). No statistically significant relationship was found between the preoperative neuropathies and the AT distance (Table 3).

**Table 3:** Relationship between the preoperative acromion-greater tuberosity distance on X-ray and the presence of injury on each branch of the nerves (A-T, acromion-greater tuberosity distance; SD, standard deviation)

Nerve	Injury	A-T (X-ray/mm)	SD	*p*
Anterior branch, axillary nerve	Normal	6.7	4.9	.830
	Chronic injury	8	6.4	
	Disuse injury	7.5	4	
Middle branch, axillary nerve	Normal	6.8	5.3	.500
	Chronic injury	7.2	4.0	
	Disuse injury	10.8	7.9	
Posterior branch, axillary nerve	Normal	7.8	5.2	.733
	Chronic injury	5	5.1	
	Disuse injury	7.5	5.5	
Supraspinatus branch	Normal	7.4	5.1	.089
	Chronic injury	3.6	3.3	
	Disuse injury	11.3	6.3	
Infraspinatus branch	Normal	6.9	5.4	.758
	Chronic injury	7.8	5.3	
	Disuse injury	8.9	5.7	

### Correlation between final functional outcome and preoperative electrodiagnostic study

There were no statistically significant differences in VAS, ROM, and rCMS between the patients without and with preoperative neurological injury: flexion 82 ± 8 vs. 72 ± 21, p 0.42; abduction 79 ± 10 vs. 71 ± 16, *p* 0.39; rCMS 38 ± 9.5 vs. 35 ± 8.2, *p* 0.28.

Regardless of pre-surgery neuropathy, a statistically significant improvement in VAS, ROM (flexion and abduction) and rCMS was present after RSA surgery. When comparing the patterns of improvement between patients with chronic injury, disuse injury, and without injury of each nerve branch, a generalised pattern of improvement was obtained without differences between the subgroups *p* > 0.05 (Table 4).

**Table 4:** Median pain and function results before surgery and 24 months after surgery (VAS, visual analogue scale; rCMS, relative Constant-Murley Score; preOp, preoperative; postOp, postoperative; SD, standard deviation; p.intra, *p* value for the difference between the values for each branch and type of injury before and after surgery; p.inter, *p* value for the difference in the improvement between the three injury types of each branch)

Nerve	Injury	VAS						rCMS						FLEXION						ABDUCTION					
		preOp	SD	postOp	SD	*p*.intra	*p*.inter	preOp	SD	postOp	SD	*p*.intra	*p*.inter	preOp	SD	postOp	SD	*p*.intra	*p*.inter	preOp	SD	postOp	SD	*p*.intra	*p*.inter
Anterior, axillary	Normal	8.5	1.2	2.1	2.6	*p* < .001	.367	38.5	8.0	83.6	23.8	*p* < .001	.544	80	32.1	123	34.1	*p* < .001	.593	67	23–8	113	34.0	*p* < .001	.954
	Chronic	8.2	1.0	1.9	1.6	*p* < .001		36.8	10.1	90.3	9.7	*p* < .001		71	23.8	130	32.5	*p* < .001		70	19.5	118	35.4	.001	
	Disuse	6.7	1.2	2.5	.5	.004		49.9	17.6	92.7	14.4	.001		95	10.0	142	30.9	.025		90	.0	130	31.6	.068	
Middle, axillary	Normal	8.3	1.3	2.4	2.3	*p* < .001	.777	40.0	11.4	84.3	20.9	*p* < .001	.352	85	26.0	122	33.1	*p* < .001	.027	73	21.7	154	32.1	*p* < .001	.270
	Chronic	8.1	.9	1.3	1.2	*p* < .001		34.1	9.4	91.1	10.4	*p* < .001		56	30.6	133	34.4	*p* < .001		62	25.2	110	36.8	.007	
	Disuse	7.3	.5	1.3	1.5	.001		44.9	2.7	100	.0	*p* < .001		76	11.5	156	5.7	.001		75	13.2	156	5.7	.002	
Posterior, axillary	Normal	8.3	1.4	3.0	3.2	*p* < .001	.765	39.1	16.9	77.0	29.4	*p* < .001	.290	90	37.2	112	38.8	.104	.057	71	23.4	106	36.6	.001	.331
	Chronic	8.3	1.2	1.6	2.0	*p* < .001		47.4	1.2	95.0	8.5	.001		70	26.4	143	37.8	.002		63	28.8	140	43.5	.001	
	Disuse	8.0	1.2	1.8	1.5	*p* < .001		38.2	11.2	90.0	12.8	*p* < .001		76	23.9	132	28.9	*p* < .001		72	20.9	118	30.8	*p* < .001	
Supraspinatus	Normal	6.7	1.1	2.1	2.4	*p* < .001	.463	36.8	8.7	85.8	21	*p* < .001	.868	77	27.9	127	33.8	*p* < .001	.438	67	23.5	115	33.0	*p* < .001	.274
	Chronic	8.5	2.8	1.8	1.4	*p* < .001		39.4	8.5	82.8	10.9	*p* < .001		79	36.8	110	28.2	.091		80	12.2	100	36.7	.27	
	Disuse	8.2	1.2	2.2	.9	.003		53.2	14.8	100	.0	*p* < .001		90	16.3	155	12.9	.003		85	10.0	145	17.3	.006	
Infraspinatus	Normal	8.2	1.0	2.1	2.3	*p* < .001	.736	37.7	9.0	85.8	21.2	*p* < .001	.988	77	30.5	131	33.8	*p* < .001	.555	70	22.3	120	36.5	*p* < .001	.496
	Chronic	8.0	1.0	3.0	1.0	*p* < .001		41.4	8.2	88.6	9.8	.002		86	5.7	120	34.6	.159		83	5.7	103	15.2	.407	
	Disuse	8.0	2.1	1.4	1.1	*p* < .001		45.9	17.5	92.4	7.5	*p* < .001		81	24.5	118	30.3	.048		71	25.1	114	27.9	.027	

At final follow-up (24 months), the patients with preoperative injured branches, which did not show improvement in the postoperative electrodiagnostic study at six months, tended to present lower VAS and rCMS values compared to the patients with nerve injuries that improved.

The mean VAS score in the patients whose electrodiagnostic study improved was 1.44 vs 2.66 in those in whom it did not (*p* 0.14). The difference between the mean rCMS of those who improved was 91.6 vs. 89.04 in those who did not improve (*p* 0.27). These lower functional results are more evident for the VAS score in the SSN injuries at final follow-up (Table 5).

**Table 5:** VAS and rCMS for the different electrodiagnostic results (VAS, visual analogue scale; rCMS, relative Constant-Murley Score; Med., median; SD, standard deviation; imprv., improvement). Median values for VAS and rCMS depending on the previous normal or injured state of each branch and its electrodiagnostic improvement or otherwise

	Nerve injury improvement	Anterior axillary			Middle axillary			Posterior axillary			Supraspinatus			Infraspinatus		
		Med	SD	*P*	Med	SD	*P*	Med	SD	*P*	Med	SD	*P*	Med	SD	*p*
VAS	Normal	2.1	2.7	0.7	2.1	2.3	.4	3.0	3.2	.1	2.1	2.4	.6	1.7	2.3	.4
	Improvement	1.5	.9		1.4	1.2		1.4	1.2		1.7	1.0		1.2	.9	
	No improv	2.3	1.9		1.0	1.4		2.5	1.9		4.0	0		3.5	.7	
rCMS	Normal	85.3	22.8	.6	84.2	21.4	.4	77.0	29.4	.2	58.8	21.0	0.7	88.1	19.2	.9
	Improvement	93.1	12.0		94.0	10.3		91.9	11.3		91.1	12.5		87.9	8.2	
	No improv	85.3	8.4		94.5	7.7		87.8	14.9		85.1	0		92.5	10.4	

## Discussion

Several studies have analysed neurological complications during [6, 7] or after [8–10]. RSA, but none focus on the patient’s preoperative neurological situation. This is the first study to prospectively analyse, by electrodiagnostic study, injuries to SSN and AN in RCTA, their evolution and clinical impact after RSA.

The prevalence of preoperative nerve injuries in RCTA in the present study was 83.9% (77% for the AN and 45% for the SSN). Comparison with other studies is difficult, since previous papers have not clearly defined the electrodiagnostic criteria for nerve injuries. Furthermore, the prevalence of SSN has been reported to be between 8 and 42% [10].

Vad et al. [4]. found a prevalence of SSN injuries of 8%. Collin et al. [11] found 2%. Costourus et al. [12] found 38% of isolated SSN injuries in patients with massive RCT. Mallon et al. [13] reported that all the patients had SSN injuries. Boykin et al. [14] confirmed by electrodiagnostic study 42% SSN injuries. Additionally, several studies have published a prevalence of between 20 and 33% of SSN injuries associated with elite athletes [2, 15, 16]. In relation to AN injury, the literature is scarce and limited to the association with a traumatic event [3]. Precise prevalence is unknown. Vad et al. [4] describe a prevalence in massive RCT of 16% with 50% of patients having a trauma history. Costouros et al. [12] also describe a prevalence of 15% AN injury, but Laderman et al. [17] do not report any axillary nerve injury comparing pre- and postoperative incidence in anatomic and RSA.

**Fig. 4:**
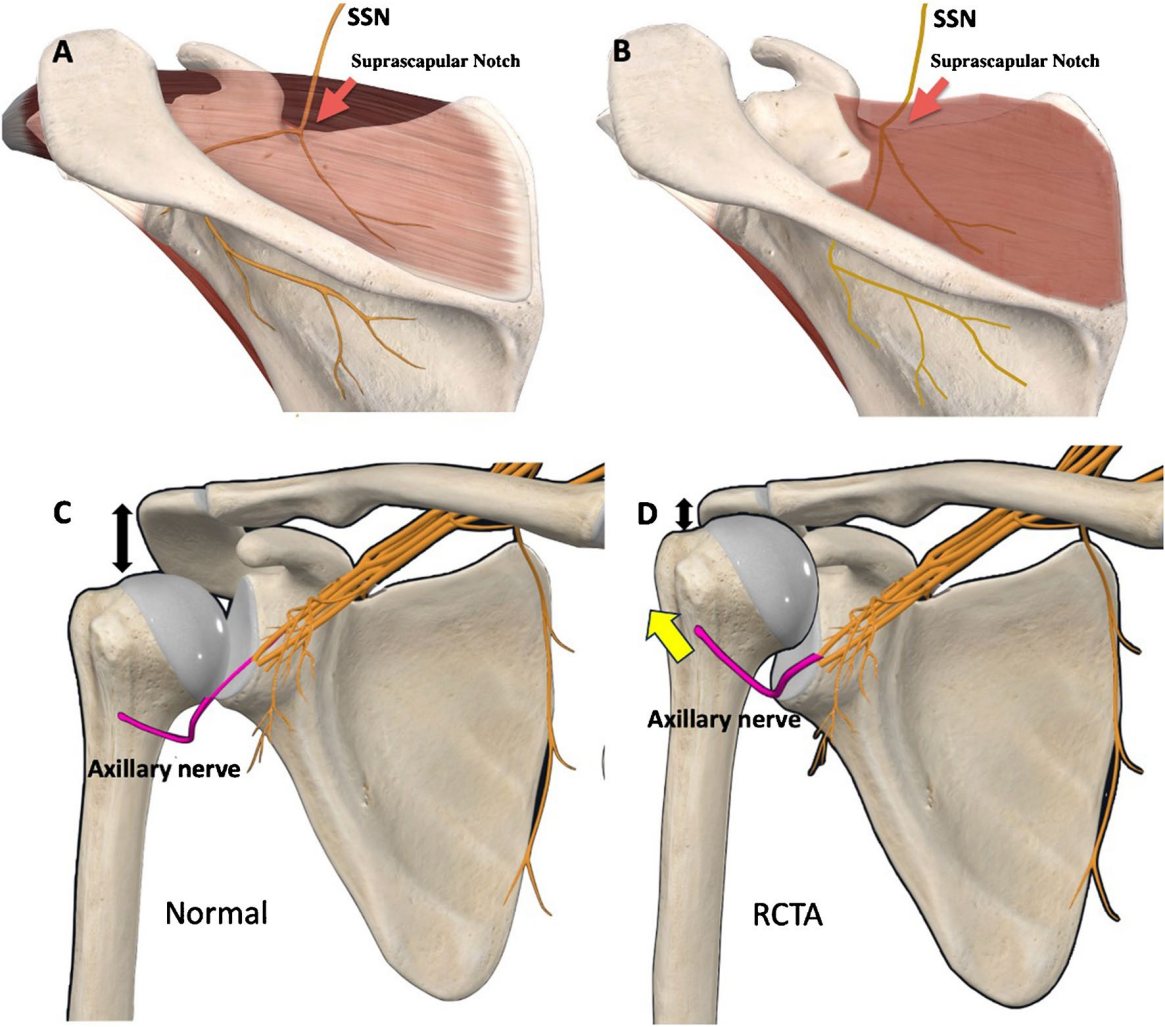
Preoperative nerve injuries. **A**) modification of the course of the suprascapular nerve when passing through the suprascapular notch **B**) Overstretching of the AN over the humeral head during shoulder anterosuperior escape may cause elongation of the free portion

With respect to the aetiopathogenesis of the AN injury, it has been hypothesised that it can be the cause or consequence of RCTs [4]. One hypothesis suggests that an idiopathic plexopathy could cause capsular weakness that would promote a RCT. However, there is not yet enough scientific evidence to support this association. It is much more likely that modified kinematics caused by a massive RCTA would be the cause of a chronic AN traction injury, mostly in patients with long-term evolution of RCTAs, like those included in this series, who have chronic attrition rupture and an anterosuperior escape (Fig. 4). The free course of the AN is relatively short. It is connected to the teres minor usually via a single branch but is anchored to the deltoid by numerous branches [18]. Overstretching of the AN over the humeral head during shoulder anterosuperior escape may cause elongation of the free portion. To establish whether there is any correlation between the level of humeral head elevation and the presence of a nerve injury, we recorded the subacromial distance measuring the preoperative radiographic acromion-greater tuberosity distance, but we did not find any statistically significant relationship between these variables.

Additionally, there is a high preoperative prevalence of disuse injuries. The authors relate this injury to the characteristics of the patients with a high mean age (78 years old and more than 50% over 80 years old) and with a pseudoparalytic shoulder that had developed over several years. The disuse injuries could be, therefore, the consequence of lack of muscle activation. The most frequent AN disuse injury was in the posterior branch of the deltoid muscle. We do not have a clear explanation for this. The free course of the nerve is relatively short, which could result in more traction of the posterior branch.

The most widely accepted theory for the origin of preoperative SSN injuries is the modification of the course of the nerve when passing through the suprascapular notch (Fig. 4), which can be caused by muscle retraction (as occurs in RCTA) with excessive traction on the nerve. Several authors have suggested the presence of traction injuries [19–21]. What may vary is the origin of the first motor branch of the nerve, and there are anatomical variants where it can branch out proximal or distal to the notch. Mallon et al. [13] proposed that when the rotator cuff tear develops slowly, the nerve adapts progressively and is able to resist the traction, but if the rotator cuff tear and the retraction occur acutely, it is very likely to cause a nerve injury. Finally, another theory for preoperative SSN injuries is the alteration of the kinematics of the shoulder girdle in the absence of rotator cuff, which could cause excessive scapular mobility [22] and subsequent nerve traction. In the present study both branches were similarly affected in the preoperative electrodiagnostic study, which could be more in line with the aforementioned theory of traction injury at the scapular notch.

With respect to the preoperative clinical impact (rCMS, ROM, and VAS) of these injuries, there were no differences between patients with or without nerve injury, and also, there were no differences based on the type of injury. This simply means that it is difficult to differentiate clinically between a cuff tear, a neurological injury, or the coexistence of both pathologies.

Post-surgery, all preoperative AN injuries evolved favourably with no differences according to type of injury or affected branch. Postoperative clinical improvement of diagnosed neurological injuries has been previously described by other authors [11, 23, 24] at a mean time of 7.4 months (the AN recovered at a mean time of 3.4 months, and the SSN at a mean time of 9 months). In our electrodiagnostic study, 69% improved within six months after surgery.

A disuse injury seems to be related to lack of activation of the deltoid. Probably, this etiopathogenesis explains the postoperative improvement, due to the increase in the recruitment of deltoid fibres and, therefore, their activation (anterior branch improved at six months in 46.7%, middle branch in 77.8%, and posterior branch in 69.2%). This leads us to support Ladermann’s proposal [17] that in certain circumstances, an inappropriate deltoid function is not an absolute contraindication to the use of RSA.

With respect to the SSN, 89% of the supraspinatus branch injuries improved in the electrodiagnostic study. The authors believe that the improvement could be related to the medialisation of the centre of rotation caused by the RSA. However, 50% of the infraspinatus branches did not improve. The clinical situation (VAS, rCMS) of patients with chronic preoperative injuries and no improvement in the second postoperative electrodiagnostic study at six months was clearly inferior. Although SSN has been historically considered primarily a motor nerve, the poor results of recovery of injury to it could be related to recent data suggesting that it would provide up to 70% of the sensitivity of the shoulder. Besides, the target musculature of the SSN is probably more difficult to compensate than other motor branches during the intramuscular collateral reinnervation process [5].

## Study strengths and limitations

The major strength of this study is its prospective design. Second, all electrodiagnostic studies were performed by only one neurophysiologist expert in upper limb. Finally, surgeries were also always performed by the same team. However, the study also has important limitations: this is a relatively small sample of patients and as such we may not have had adequate statistical power to show significant differences. We just analysed the presence of preoperative nerve injuries, their evolution, and their influence on RSA results. The consideration of the influence of other factors such as anaesthetic block technique, the approach, or the surgery itself that could cause acute injuries after RSA are not the object of this study.

## Disclaimer

The authors, their immediate families, and any research foundations with which they are affiliated have not received any financial payments or other benefits from any commercial entity related to the subject of this article.

## Conclusions

The frequency of axillary and suprascapular nerve injuries in preoperative rotator cuff arthropathy in reverse shoulder arthroplasty is much higher than expected, most of these preoperative injuries are reversible after surgery, with almost complete neurophysiological recovery and with little functional impact on RSA. However, those patients without neurophysiological improvement of preoperative nerve injuries 6 months after surgery have lower functional results.

## Competing interests

The authors declare no competing interests.

## Data Availability

All data generated or analysed during this study are included in this published article.
